# Data for crystallisation, dissolution and saturation temperatures of a model fuel comprising eicosane crystallising from supersaturated toluene solutions in the presence of a cold-flow improver additive

**DOI:** 10.1016/j.dib.2022.108455

**Published:** 2022-07-08

**Authors:** Peter L. Kaskiewicz, Ruth Downie, Peter J. Dowding, Neil George, Kevin J. Roberts

**Affiliations:** a School of Chemical and Process Engineering, University of Leeds, Leeds, UK; b Infineum UK Ltd, Milton Hill Business and Technology Centre, Abingdon, UK; c Syngenta UK Ltd, Jealott's Hill Business Park, Bracknell, UK

**Keywords:** Crystallization, Fuel, Additive, Solubility, Turbidity

## Abstract

The data presented in this article relates to the crystallisation of the long chain hydrocarbon eicosane (C_20_H_42_), from supersaturated toluene solutions in the absence/presence of a commercially available cold-flow improver additive (IA) at different solution treat rates. Data was collected for treat rates of 0, 0.1, 0.5, 2, 3, 5 and 10 wt% IA with respect to eicosane, with each treat rate studied over four solution concentrations. Data is collected by transmission vs. solution temperature experimental investigations and is analysed through a conventional transmission analysis route (STR) and a reanalysed route that takes into account multiple phase transformation behaviour (RRT). Average crystallisation and dissolution data is provided over a range of solution concentrations and cooling rates used under a polythermal crystallisation methodology for each analysis route. Equilibrium saturation temperature, supersolubility and metastable zone width data is also presented for each treat rate, concentration and analysis route. Laser transmission as a function of solution temperature profiles are displayed for IA crystallising from toluene solutions. This data relates to the research article: Kaskiewicz, P. L., Downie, R., Dowding, P. J., George, N. & Roberts, K. J. Influence of a Polymeric Additive on the Crystallisability and Nucleation Mechanism for the Model Fuel System of Eicosane Crystallising from Supersaturated Toluene Solutions. J. Cryst. Growth 581, (2021) 126,470. https://doi.org/10.1016/j.jcrysgro.2021.126470

## Specifications Table

**Table:** 

Subject	Chemical Engineering
Specific subject area	Crystallisation, additives and fuel
Type of data	Tables and figures
How data were acquired	In this study the data was collected from crystallisation experiments using the Technobis Crystal16 apparatus.
Data format	Raw and Analysed
Description of data collection	Solutions were heated and agitated prior to crystallisation in order to generate homogenized solutions. Each experimental run was repeated five times. Laser transmission vs. solution temperature data concerning the crystallisation and dissolution temperatures was acquired in order to calculate equilibrium saturation and solubility temperatures.
Data source location	University of Leeds, Leeds, West Yorkshire, United Kingdom 53.8067° N, 1.5550° W
Data accessibility	Data is with this article and is openly available from University of Leeds Data Repository at https://doi.org/10.5518/1172
Related research article	P.L. Kaskiewicz, R. Downie, P.J. Dowding, N. George and K.J. Roberts. Influence of a Polymeric Additive on the Crystallisability and Nucleation Mechanism for the Model Fuel System of Eicosane Crystallising from Supersaturated Toluene Solutions. Journal of Crystal Growth. 581, (2021) 126,470. https://doi.org/10.1016/j.jcrysgro.2021.126470. [1]

## Value of the Data

• Crystallisation of wax components within diesel fuel is a long-standing technological problem with very little data present within open literature on this behaviour. Therefore, data collected in relation to this, such as that presented in this article, provides much needed information for researchers working in this area.
• The mechanism and impact of cold-flow improver additives retardation of the crystallisation of wax within diesel fuel is not fundamentally well understood, therefore, data pertaining to the inhibitory effects of a cold-flow improver additive (IA) upon the crystallisation of a wax (eicosane) is invaluable for those working in this area and those undertaking research into these effects.
• Future work on further understanding and potentially controlling wax crystallisation by polymeric additives in diesel fuel can be built from this data, by either continuation of these results or via comparison of this data to either corroborate or contradict these findings, which would generate a much needed improvement in the availability and in-depth analysis of such data.
• This data provides substantial evidence that when crystallisation studies are performed for multi-component systems, especially those containing polymeric based additive, it is imperative that the raw analytical data be understood in order to generate accurate and useful data. This is highlighted through turbidity measurements within this article, which is a common technique for assessing crystallisation and dissolution processes.

## 1 Data Description

The data in this article relates to the crystallisation behaviour of eicosane from toluene solutions in the absence and presence of a commercially available cold-flow improver additive (IA) at different treat rates (additive concentrations). Turbidity (laser transmission) as a function of solution temperature data is recorded over time and at heating/cooling cycles at different constant rates (q). Analysis of transmission data is performed in two ways, with one analysis route, referred to as STR, utilising the standard transmission analysis of defining the onset a crystallisation as the point at which transmission reduces below 100% and dissolution assessed at the point at which transmission reaches 100%. The second analysis route, termed RRT, accounts for possible multi-phase transitional behaviour and assesses the transmission vs. temperature plots to determine the crystallisation and dissolution phase transformation behaviour of specific components within the multi-component solution.

**Fig. 1:**
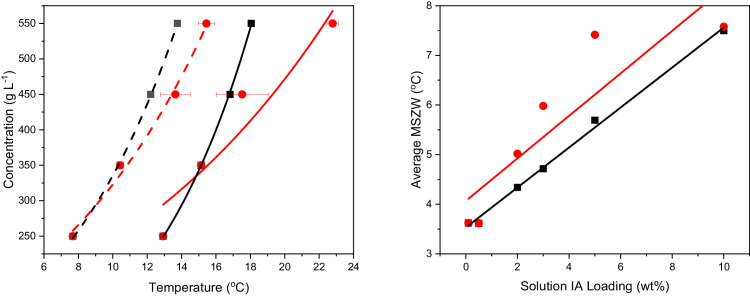
(a) MSZWs for eicosane in toluene solution with 3 wt% IA loading. Solubility is represented by solid lines and supersolubility is represented by dashed lines. Black squares represent data calculated by taking account of the impact of IA on the transmittance (RRT analysis). Red circles represent data collected through standard transmission data analysis (STR analysis). Data is fitted with simple exponentials. (b) Increase in average MSZW as a function of IA loading (treat rate). Black squares represent data calculated from the raw data analysis taking into account IA impact on transmission (RRT analysis). Red circles represent data calculated from the standard transmission analysis route (STR analysis). Both data sets are fitted with linear regressions.

**Fig. 2:**
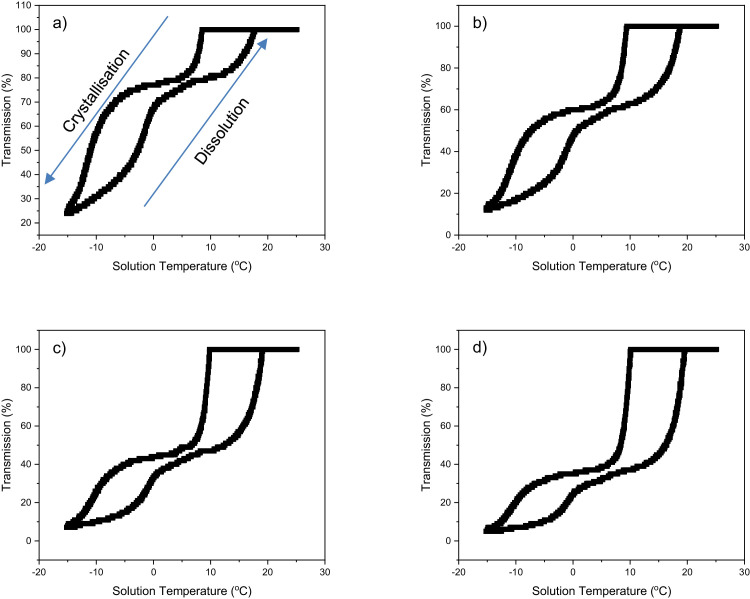
Laser transmission as a function of solution temperature for IA crystallising and dissolving from toluene solutions at a heating/cooling rate of 0.25 °C min^-1^, at four solution concentrations: (a) 70 g l^-1^, 90 g l^-1^, 110 g l^-1^, 130 g l^-1^.

Crystallisation (Tc) and dissolution (Tdiss) temperatures of eicosane crystallising from toluene solutions for each additive treat rate, at each cooling/heating rate used for the crystallisation experiments and each solution concentration are shown in Tables 1 and 2 for the RRT and STR analysed data, respectively, with 5 repeats taken for each measurement. Table 3 displays the equilibrium saturation temperatures (Te), the steady-state crystallisation temperature at the kinetic limit (Tc/l) and the steady-state metastable zone widths (MSZWs) determined for each IA solution treat rate over the full range of solution concentrations studied. Data is shown for values determined using the RRT and STR analysis routes. The impact of the analysis route taken on the determined crystallisation and dissolution temperatures is observed for the values of Te and Tc/l for an IA treat rate of 3 wt% IA in Fig. 1(a) and the MSZW behaviour for a single IA treat rate and its variation as a function of solution treat rate is shown in Fig. 1(b), for data analysed through RRT and STR. Finally, example plots of laser transmission as a function of solution temperature for IA crystallising from toluene solutions, in the absence of eicosane, at four solution concentrations and at a single heating/cooling rate are given in Fig. 2, demonstrating the crystallisation and dissolution profile behaviours of IA.

**Table 1:** Temperatures of crystallisation (Tc) and dissolution (Tdiss) as a function of heating/cooling rates [q (°C min^-1^)] and IA treat rates ranging from 0 to 10 wt%, of eicosane crystallising from toluene solutions in the absence and presence of IA at a range of solution concentrations. Data collected using the RRT analysis route. Each Tc and Tdiss measurement was repeated (#) 5 times.

0 wt% IA										0.1 wt% IA									
		Tc				Tdiss						Tc				Tdiss			
q °C min^-1^	#	250 (g l^-1^)	350 (g l^-1^)	450 (g l^-1^)	550 (g l^-1^)	250 (g l^-1^)	350 (g l^-1^)	450 (g l^-1^)	550 (g l^-1^)	q °C min^-1^	#	200 (g l^-1^)	300 (g l^-1^)	400 (g l^-1^)	500 (g l^-1^)	200 (g l^-1^)	300 (g l^-1^)	400 (g l^-1^)	500 (g l^-1^)

0.25	1	9.6	12.7	14.8	16.0	12.0	14.6	16.5	17.8	0.25	1	7.0	10.6	13.0	14.8	11.8	14.7	16.9	18.5
	2	10.0	12.6	14.5	16.0	12.1	14.9	16.3	18.2		2	7.3	10.5	13.1	14.9	11.8	14.8	17.1	18.7
	3	9.5	12.5	14.3	16.2	11.8	14.5	16.5	17.9		3	7.4	10.7	13.2	14.8	11.8	14.8	17.0	18.6
	4	9.7	12.6	15.1	16.3	12.0	14.6	16.5	18.0		4	7.2	10.7	13.0	14.6	11.8	14.9	17.0	18.7
	5	10.0	12.7	14.7	16.0	12.0	14.6	16.6	18.0		5	7.3	10.6	13.1	15.0	11.8	14.7	16.9	18.4
1	1	9.2	12.1	13.9	15.1	13.2	14.6	18.2	20.1	1	1	6.4	10.2	12.7	14.1	12.7	16.5	19.0	20.5
	2	9.4	12.1	13.7	15.9	12.5	16.4	18.3	20.3		2	6.7	9.7	12.5	14.2	12.9	16.7	19.0	20.8
	3	9.0	12.0	13.9	15.0	13.3	15.7	18.2	20.5		3	6.7	10.2	12.2	14.4	12.9	16.5	18.9	20.4
	4	9.0	12.0	13.7	15.1	13.6	15.2	18.0	20.6		4	7.1	10.0	12.2	14.3	13.2	16.7	19.0	20.8
	5	9.4	12.2	13.7	15.6	12.3	15.6	18.6	20.3		5	6.7	10.2	12.4	14.1	12.8	16.7	19.1	20.6
2	1	7.9	11.3	13.0	14.5	15.8	18.0	20.4	21.8	2	1	5.7	9.3	12.1	13.6	14.2	17.8	20.6	22.4
	2	8.4	11.0	13.0	14.6	15.0	19.2	19.9	21.6		2	5.6	9.0	11.7	–	14.4	17.7	20.5	22.4
	3	8.6	11.5	13.5	15.1	15.2	18.8	20.3	21.7		3	6.4	9.0	11.8	13.7	14.1	17.6	20.6	22.6
	4	8.4	11.3	13.4	15.0	14.4	17.9	20.3	21.6		4	6.3	9.5	11.7	13.4	14.2	18.0	20.6	22.5
	5	8.2	11.1	13.2	14.7	15.0	18.6	19.9	21.8		5	6.3	9.1	11.8	13.4	14.5	17.6	20.6	22.6
3.2	1	7.7	10.4	12.7	13.9	17.4	20.0	21.8	24.0	3.2	1	5.2	8.2	10.9	12.3	16.1	20.2	22.7	25.1
	2	8.0	10.2	12.8	13.9	18.0	20.3	22.2	24.1		2	5.1	8.6	11.1	12.8	16.1	19.9	22.7	25.4
	3	7.3	10.3	12.8	14.2	17.3	20.2	22.0	24.5		3	5.2	8.7	–	13.2	16.3	20.2	22.7	25.2
	4	7.5	10.3	12.8	14.0	16.8	21.5	23.2	24.0		4	5.3	8.5	10.9	12.5	16.0	20.1	22.8	25.5
	5	7.4	10.5	12.9	14.1	16.8	20.3	22.1	24.4		5	5.1	8.5	11.3	12.8	16.0	20.3	22.9	25.1

0.5 wt% IA										2 wt% IA									
0.25	1	8.0	11.7	13.8	15.1	13.2	15.6	17.5	18.9	0.25	1	8.1	10.9	12.6	14.6	13.2	15.5	17.2	18.6
	2	–	11.7	13.5	14.4	13.5	15.8	17.7	19.2		2	7.7	10.6	12.5	14.0	13.3	15.5	17.4	18.6
	3	8.2	11.9	13.6	14.8	13.1	15.8	17.6	19.2		3	7.8	10.7	12.6	14.1	13.1	15.3	17.2	18.5
	4	7.7	11.6	13.7	15.0	13.1	15.5	17.5	19.0		4	7.7	10.8	12.5	14.2	13.1	15.5	17.2	18.5
	5	8.1	11.8	13.6	15.1	13.2	15.7	17.6	19.0		5	7.8	10.7	12.5	14.3	13.1	15.4	17.2	18.4
1	1	8.3	10.9	12.9	14.5	14.7	17.5	19.5	21.1	1	1	7.7	10.2	12.0	13.5	14.2	16.9	18.9	20.5
	2	8.5	11.2	13.1	14.8	14.8	17.9	19.9	21.4		2	7.7	10.4	12.3	13.7	14.7	17.1	19.2	20.8
	3	8.3	10.7	12.7	14.3	14.9	17.7	19.7	21.2		3	7.1	9.8	12.0	13.1	14.5	16.9	19.2	20.8
	4	8.5	11.0	12.9	14.3	15.1	17.7	19.8	21.4		4	7.4	9.8	12.0	13.3	14.8	16.9	19.2	20.8
	5	8.2	11.0	12.9	14.5	14.7	17.7	19.5	21.1		5	7.7	10.2	12.0	13.4	14.7	16.9	19.0	20.5
2	1	7.1	10.3	12.5	13.7	16.3	19.0	21.2	23.4	2	1	6.6	9.3	11.4	12.6	16.1	18.6	20.9	22.7
	2	7.5	10.5	12.6	13.6	16.3	19.0	21.4	23.6		2	6.2	9.4	11.3	12.7	16.0	18.8	20.8	22.9
	3	7.9	10.8	12.5	13.8	16.4	19.0	21.2	23.3		3	6.8	9.7	11.8	12.9	16.0	18.5	20.8	22.7
	4	7.4	10.7	12.9	14.0	16.2	19.0	21.2	23.5		4	6.7	9.6	11.7	12.8	15.9	18.6	20.8	22.8
	5	7.6	10.5	12.4	13.8	16.6	19.2	21.4	23.6		5	6.4	9.5	11.5	12.9	16.1	18.8	21.0	22.8
3.2	1	6.0	9.7	11.1	13.0	18.5	22.0	24.3	27.0	3.2	1	5.7	8.8	10.7	12.1	18.1	20.6	23.1	24.9
	2	6.5	9.7	11.9	13.0	19.3	22.0	24.5	26.7		2	5.9	8.8	10.9	12.1	18.0	20.5	23.4	24.9
	3	6.4	9.9	12.0	13.0	18.6	21.9	24.4	26.4		3	6.2	9.0	10.9	12.0	18.1	20.7	23.1	24.9
	4	6.9	9.4	12.1	13.0	19.5	21.9	24.4	26.6		4	6.0	8.9	10.7	12.1	18.1	20.6	23.3	24.8
	5	6.6	9.7	12.0	13.0	19.1	22.1	24.4	26.8		5	5.8	8.8	10.8	12.0	17.8	20.6	23.1	24.9
3 wt% IA										5 wt% IA									
0.25	1	7.4	10.3	12.2	13.8	13.2	15.5	17.2	18.5	0.25	1	6.4	9.2	11.2	12.6	13.1	15.5	17.0	18.4
	2	7.4	10.2	12.0	13.5	13.3	15.5	17.3	18.5		2	6.2	9.0	11.0	12.4	13.3	15.5	17.1	18.5
	3	7.6	10.2	12.2	13.7	13.2	15.5	17.2	18.5		3	6.7	9.3	11.2	12.6	13.2	15.5	17.2	18.5
	4	7.4	10.2	12.2	13.7	13.3	15.5	17.2	18.5		4	6.6	9.2	11.2	12.6	13.1	15.4	17.0	18.4
	5	7.6	10.4	12.2	13.7	13.3	15.5	17.2	18.5		5	6.8	9.3	11.2	12.7	13.1	15.3	17.0	18.4
1	1	6.9	9.6	11.6	12.7	14.5	16.8	18.9	20.1	1	1	6.2	8.8	10.4	10.0	14.3	17.0	18.6	21.5
	2	7.3	9.7	11.8	13.3	14.9	17.1	19.3	20.4		2	6.2	8.7	10.6	11.4	14.7	16.9	18.8	22.5
	3	6.8	9.6	11.4	12.8	14.5	16.9	19.0	20.4		3	6.0	8.5	10.6	8.2	14.4	17.1	18.6	21.4
	4	6.8	9.7	11.6	12.8	14.7	17.1	18.9	20.5		4	6.0	8.5	10.4	8.1	14.4	17.1	18.8	21.7
	5	7.1	9.8	11.6	13.1	14.6	16.9	19.0	20.4		5	6.0	8.6	10.6	8.4	14.4	16.8	18.6	21.3
2	1	5.5	9.0	10.9	12.1	16.4	18.5	20.7	22.4	2	1	5.1	8.0	10.1	9.4	16.1	18.6	20.8	24.0
	2	6.0	9.0	10.7	12.3	16.3	18.5	20.8	22.4		2	5.3	8.2	10.1	10.3	15.7	18.7	21.0	24.2
	3	6.2	9.2	11.0	12.6	16.1	18.5	20.6	22.2		3	5.1	8.4	10.3	9.0	15.8	18.4	20.8	23.8
	4	5.8	9.2	11.1	12.5	16.1	18.5	20.7	22.2		4	5.5	8.3	10.2	8.6	15.8	18.4	20.8	23.7
	5	5.7	9.1	10.9	12.3	16.4	18.6	20.8	22.5		5	5.2	8.2	10.2	8.0	16.0	18.6	20.8	23.9
3.2	1	5.1	8.3	10.5	11.7	18.1	20.6	23.2	24.8	3.2	1	5.0	7.7	9.7	10.9	18.8	21.3	23.5	24.9
	2	5.5	8.1	10.4	11.6	18.1	20.6	23.2	25.0		2	5.1	7.6	9.8	10.7	18.4	21.4	23.4	24.8
	3	5.1	8.3	10.2	11.6	18.1	20.6	23.3	25.1		3	–	7.7	9.1	10.7	18.6	21.5	23.4	24.8
	4	5.2	8.4	10.3	11.6	18.4	20.8	23.2	24.9		4	4.9	7.9	9.4	10.7	18.7	21.5	23.5	24.9
	5	5.3	8.5	10.5	11.8	18.2	20.8	23.2	25.1		5	5.1	7.8	9.7	10.9	18.5	21.1	–	24.8

10 wt% IA																			
0.25	1	4.4	7.2	9.1	10.5	12.9	15.2	16.7	18.1										
	2	4.3	7.1	8.9	10.3	12.9	15.3	16.8	18.2										
	3	4.4	7.2	9.0	10.5	12.7	15.1	16.7	18.1										
	4	4.3	7.1	8.9	10.4	12.7	15.1	16.8	18.1										
	5	4.3	7.2	8.9	10.4	12.7	15.1	16.7	18.1										
1	1	3.8	6.8	8.3	9.6	14.1	16.7	18.2	19.6										
	2	3.7	6.8	8.6	9.8	14.3	16.9	18.3	19.8										
	3	3.3	6.4	8.3	9.4	14.1	16.7	18.4	19.8										
	4	3.7	6.5	8.3	9.6	14.3	16.8	18.4	19.7										
	5	3.8	6.5	8.3	9.6	14.1	16.8	18.1	19.8										
2	1	3.0	6.2	7.8	8.9	15.1	18.1	20.4	22.4										
	2	3.1	6.2	8.0	9.1	15.0	17.8	20.4	22.4										
	3	3.4	6.5	8.2	9.2	14.8	17.8	20.3	22.9										
	4	3.4	6.3	8.1	9.3	15.0	17.8	20.2	22.9										
	5	3.2	6.5	8.0	9.2	15.1	17.9	20.2	22.5										
3.2	1	2.2	5.5	7.6	8.5	18.4	20.4	21.8	24.5										
	2	1.9	5.5	7.5	8.6	18.2	20.6	22.8	24.7										
	3	2.2	5.6	7.5	8.7	18.2	20.4	21.8	24.4										
	4	2.2	5.4	7.4	8.6	18.2	20.4	22.7	24.3										
	5	2.0	5.7	7.7	8.6	17.9	20.1	22.0	24.4

**Table 2:** Temperatures of crystallisation (Tc) and dissolution (Tdiss) as a function of heating/cooling rates [q (°C min^-1^)] and IA treat rates ranging from 0 to 10 wt%, of eicosane crystallising from toluene solutions in the absence and presence of IA at a range of solution concentrations. Data collected using the STR analysis route. Each Tc and Tdiss measurement was repeated (#) 5 times. Data collected for treat rates of 0–0.5 wt% IA was the same as the RRT analysis route and so is omitted from this table.

2 wt% IA										3 wt% IA									
		Tc				Tdiss						Tc				Tdiss			
q °C min^-1^	#	250 (g l^-1^)	350 (g l^-1^)	450 (g l^-1^)	550 (g l^-1^)	250 (g l^-1^)	350 (g l^-1^)	450 (g l^-1^)	550 (g l^-1^)	q °C min^-1^	#	250 (g l^-1^)	350 (g l^-1^)	450 (g l^-1^)	550 (g l^-1^)	250 (g l^-1^)	350 (g l^-1^)	450 (g l^-1^)	550 (g l^-1^)
0.25	1	8.1	10.9	12.6	14.6	13.1	15.5	17.1	18.4	0.25	1	7.4	10.3	14.3	15.6	13.2	15.5	21.7	22.9
	2	7.7	10.6	12.5	14.0	13.3	15.5	17.2	18.5		2	7.4	10.2	14.2	15.5	13.3	15.5	21.9	23.1
	3	7.8	10.7	12.6	14.1	12.9	15.3	17.1	18.3		3	7.6	10.2	14.1	15.5	13.2	15.5	22.0	23.3
	4	7.7	10.8	12.5	14.2	12.9	15.4	17.1	18.5		4	7.4	10.2	14.2	15.5	13.3	15.5	21.9	23.1
	5	7.8	10.7	12.5	14.3	13.0	15.3	17.1	18.4		5	7.6	10.4	14.2	15.6	13.3	15.5	21.8	23.1
1	1	7.7	10.2	12.0	13.5	14.1	16.8	18.9	20.5	1	1	6.9	9.6	11.6	13.5	14.5	16.8	20.1	23.4
	2	7.7	10.4	12.3	13.7	14.6	17.1	19.2	20.8		2	7.3	9.7	11.8	14.1	14.9	17.1	19.3	23.5
	3	7.1	9.8	12.0	13.1	14.5	16.8	19.0	20.5		3	6.8	9.6	11.4	13.7	14.5	16.9	19.2	23.4
	4	7.4	9.8	12.0	13.3	14.5	16.9	19.2	20.8		4	6.8	9.7	11.6	13.7	14.7	17.1	18.9	23.6
	5	7.7	10.2	12.0	13.4	14.3	16.8	19.0	20.6		5	7.1	9.8	11.6	13.9	14.6	16.9	19.7	23.5
2	1	6.6	9.3	11.4	12.6	16.1	18.6	20.9	22.7	2	1	5.5	9.0	10.9	12.5	16.4	18.5	20.7	23.7
	2	6.2	9.4	11.3	12.7	16.0	18.8	20.8	22.9		2	6.0	9.0	10.7	12.5	16.3	18.5	20.8	23.5
	3	6.8	9.7	11.8	12.9	16.0	18.5	20.8	22.7		3	6.2	9.2	11.0	12.6	16.1	18.5	20.6	23.6
	4	6.7	9.6	11.7	12.8	15.9	18.6	20.8	22.8		4	5.8	9.2	11.1	12.7	16.1	18.5	20.7	23.5
	5	6.4	9.5	11.5	12.9	16.1	18.8	21.0	22.8		5	5.7	9.1	10.9	12.4	16.4	18.6	20.8	23.6
3.2	1	5.7	8.8	10.7	12.1	18.1	20.6	23.1	24.9	3.2	1	5.1	8.3	10.5	11.7	18.1	20.6	23.2	24.8
	2	5.9	8.8	10.9	12.1	18.0	20.5	23.4	24.9		2	5.5	8.1	10.4	11.6	18.1	20.6	23.2	25.0
	3	6.2	9.0	10.9	12.0	18.1	20.7	23.1	24.9		3	5.1	8.3	10.2	11.6	18.1	20.6	23.3	25.1
	4	6.0	8.9	10.7	12.1	18.1	20.6	23.3	24.8		4	5.2	8.4	10.3	11.6	18.4	20.8	23.2	24.9
	5	5.8	8.8	10.8	12.0	17.8	20.6	23.1	24.9		5	5.3	8.5	10.5	11.8	18.2	20.8	23.2	25.1

5 wt% IA										10 wt% IA									
0.25	1	6.4	12.9	14.7	15.8	13.1	20.9	22.4	23.4	0.25	1	11.6	13.5	15.2	16.4	19.6	21.5	22.7	24.0
	2	6.2	12.7	14.5	15.7	13.3	20.9	22.6	23.7		2	11.3	13.3	14.9	16.1	19.9	21.7	22.8	24.2
	3	6.7	12.9	14.7	15.8	13.3	20.9	22.6	23.7		3	11.5	13.4	15.0	16.2	19.7	21.5	22.6	24.0
	4	6.7	12.8	14.7	15.8	13.1	20.9	22.4	23.6		4	11.4	13.3	15.0	16.1	19.8	21.6	22.6	24.0
	5	6.8	12.9	14.7	15.8	13.1	20.9	22.5	23.6		5	11.5	13.3	15.0	16.2	19.8	21.5	22.7	24.0
1	1	6.2	9.8	12.6	13.9	14.3	19.9	22.8	24.6	1	1	4.2	11.8	13.1	14.4	15.4	21.7	23.1	24.3
	2	6.2	8.7	13.0	14.1	14.7	–	22.8	24.9		2	3.7	11.8	13.5	14.8	14.3	21.7	23.2	24.4
	3	6.0	8.5	12.7	13.6	14.4	18.4	22.7	24.6		3	3.6	11.6	13.1	14.3	14.3	21.7	23.0	24.2
	4	6.0	8.5	12.6	13.7	14.5	19.2	22.9	24.8		4	3.7	11.7	13.0	14.3	14.9	21.7	23.1	24.6
	5	6.0	8.6	12.7	13.9	14.4	19.5	22.7	24.6		5	3.8	11.8	13.1	14.5	15.1	21.6	23.1	24.3
2	1	5.1	8.0	11.7	12.7	15.9	18.6	22.6	25.2	2	1	3.0	10.5	12.3	13.4	15.6	21.6	23.2	24.9
	2	5.3	8.2	11.8	12.7	15.7	18.7	22.6	25.2		2	3.4	10.7	12.3	13.4	15.5	21.6	23.5	25.1
	3	5.1	8.4	11.8	12.9	15.8	18.4	22.6	24.9		3	3.4	11.0	12.5	13.6	15.7	21.4	23.2	24.8
	4	5.5	8.3	11.7	12.8	16.4	18.4	22.4	25.0		4	3.4	10.7	12.5	13.4	15.8	21.3	23.1	24.8
	5	5.2	8.2	11.5	12.7	16.0	18.6	22.7	25.4		5	3.2	10.8	12.3	13.3	15.3	21.6	23.5	25.1
3.2	1	5.0	7.7	10.5	12.1	18.8	21.3	24.5	25.7	3.2	1	2.2	9.8	11.5	12.6	20.6	22.9	24.6	26.5
	2	5.1	7.6	10.7	12.2	18.4	21.4	24.3	25.6		2	1.9	9.7	11.4	12.7	20.9	22.8	24.8	26.5
	3	4.1	7.7	10.6	12.1	19.0	21.5	24.2	25.7		3	2.2	9.7	11.5	12.5	20.8	22.9	24.8	26.7
	4	4.9	8.1	10.7	12.0	18.7	21.5	24.4	25.8		4	2.2	9.7	11.5	12.5	20.4	22.9	24.8	26.5
	5	5.1	7.8	10.8	12.3	18.5	21.1	24.4	25.8		5	2.0	9.8	11.5	12.5	20.7	22.8	24.8	26.5

**Table 3:** Solubility ($T_{e}$) and supersolubility ($T_{c,l}$) data of eicosane in toluene solution with different loadings of IA, alongside calculated steady-state MSZW over four concentrations (200–500 g l^-1^ for 0.1 wt% IA solution and 250–550 g l^-1^ for all other solutions). Data was determined by RRT and STR from the data presented in Tables 1 and 2, respectively. Modified from Journal of Crystal Growth 581 (2021) 126,470. [1].

Conc. (g l^-1^)	$T_{c,l}$ ( °C)	$T_{e}$ ( °C)	MSZW ( °C)	Conc. (g l^-1^)	$T_{c,l}$ ( °C)	$T_{e}$ ( °C)	MSZW ( °C)	Conc. (g l^-1^)	$T_{c,l}$ ( °C)	$T_{e}$ ( °C)	MSZW ( °C)
0 wt% IA				0.1 wt% IA				0.5 wt% IA			
250	9.9	11.4	1.5	200	7.4	11.4	4.0	250	8.7	12.8	4.1
350	12.8	13.9	1.1	300	10.8	14.5	3.7	350	11.8	15.3	3.5
450	14.9	15.8	0.9	400	13.2	16.8	3.6	450	13.7	17.1	3.4
550	16.6	17.7	1.1	500	15.0	18.2	3.2	550	15.1	18.5	3.4

2 wt% IA				3 wt% IA				5 wt% IA			
STR				STR				STR			
250	8.0	12.8	4.8	250	7.7	12.9	5.2	250	6.6	12.6	6.0
350	10.8	15.1	4.3	350	10.4	15.1	4.7	350	11.9	19.7	7.8
450	12.6	17.4	4.8	450	13.6	20.3	6.6	450	14.5	22.3	7.7
550	14.5	20.7	6.2	550	15.4	22.8	7.3	550	15.6	23.7	8.1

RRT				RRT				RRT			
250	8.0	12.8	4.8	250	7.7	12.9	5.2	250	6.6	12.6	6.0
350	10.8	15.1	4.3	350	10.4	15.1	4.7	350	9.2	14.9	5.7
450	12.6	16.9	4.3	450	12.2	16.8	4.6	450	11.2	16.5	5.3
550	14.3	18.3	4.0	550	13.8	18.1	4.3	550	12.3	18.1	5.7

10 wt% IA											
STR											
250	9.6	16.8	7.2								
350	13.3	21.3	8.0								
450	14.8	22.4	7.6								
550	16.1	23.6	7.5								
RRT											
250	4.5	12.2	7.8								
350	7.2	14.8	7.6								
450	9.0	16.4	7.4								
550	10.4	17.7	7.2

## 2 Experimental Design, Materials and Methods

### 2.1 Materials

A simple model fuel system of the long chain hydrocarbon eicosane within toluene solutions in the absence and presence of a commercially available cold-flow improver additive (IA) was chosen and is detailed in Table 4.

**Table 4:** Summary of materials used for experimental research.

Chemical Name	Formula	Synonym	Purity	MW g/mol	Supplier
Eicosane	C_30_H_42_	C_20_	99%	282.55	Sigma-Aldrich
Toluene	C_7_H_8_	N.A.	≥99.3%	92.14	Sigma-Aldrich
Fuel Additive	–	IA	–	–	Infineum Ltd.

### 2.2 Equipment

The crystallisation/dissolution experiments were carried out using the Technobis Crystal 16® system [2], which comprises of sixteen wells split into four blocks, consisting of four wells each. Each block is independently temperature controlled to allow different temperature profiles to be set simultaneously, with the use of Peltier elements and an external cooling device. The system can be run between −15 and 120 °C and condensation at cooler temperatures is stopped by a dry air purge system. Each well can hold 1.5 ml disposable glass GPC vial with a small magnetic stirrer. A laser passes through the vials in order to determine turbidity and provide information about the cloud and clear points of up to 16 solutions and turbidity data (laser transmission) is recorded as a function of the temperature.

### 2.3 Experimental Procedure

#### 2.3.1 Solution Preparation

All solutions were prepared at four solution concentrations for each IA treat rate used, with concentrations of 250, 350, 450 and 550 g l^-1^ used for IA treat rates of 0, 0.5, 2, 3, 5 and 10 wt% IA, concentrations of 200, 300, 400 and 500 g l^-1^ used for the IA treat rate of 0.1 wt% IA and concentrations of 70, 90, 110 and 130 g l^-1^ used for an IA treat rate of 100 wt% IA, providing a total of 32 solutions for this study. These concentrations represent the total solution concentration including both eicosane and IA, in that, for example, a 1 L solvent solution with a concentration of 550 g l^-1^ for an IA treat rate of 0.5 wt% IA would represent and solution with 547.25 g eicosane and 2.75 g IA, in 1 L of toluene. Solutions were prepared by weighing the solid components of eicosane and IA into 15 ml prewashed glass vials, using a spatula to transfer the solid materials into the vial, which was placed on a weighing scale that could measure with ±0.1 mg accuracy. The amount of eicosane and IA that was used depended upon the required solution concentration and IA treat rate. A Fisherbrand 500–5000 μl micropipette was used to add 5 ml toluene to each solution, to make up the required solution concentration. A magnetic stirrer bar was placed into the glass vials and the mixtures were placed on a stirrer hot plate and held at 35 °C for 30 min under constant agitation of 300 rpm, in order to form a homogeneous liquid solution. After this, 1 ml of solution was withdrawn from these vials using a Fisherbrand 100–1000 μl micropipette and was subsequently transferred into a 1.5 ml glass vial, which was placed in the Technobis Crystal16 system and a standard 7 × 2 mm magnetic stirrer was added to each vial.

#### 2.3.2 Measurement and Analysis

The 1 ml solutions were subject to different cooling and heating cycles by placing them within the blocks of the Technobis Crystal 16® system, enabling a polythermal crystallisation experiment to be performed. Each well of a block contained a different solution concentration, thereby enabling a single heating/cooling rate to be set for 4 different solution concentrations. Solutions were initially heated to 37 °C, followed by a hold period of one hour to generate homogenized solutions, before being cooled to −15 °C at a set rate, followed by another 1 h hold period, before increasing solution temperature back to 37 37 °C at the same set rate. This procedure was performed at rates (q) of 0.25, 1, 2 and 3.2 °C min^-1^ for both heating and cooling segments of profile. Solutions were agitated at 300 rpm using a 7 x 2 mm magnetic stirrer bar for the duration of the experiments. Temperature cycles were repeated 5 times in order to generate mean values for crystallisation and dissolution temperatures, with standard deviations.

Transmission data as a function of solution temperature were used to determine crystallisation and dissolution temperatures for all solutions studied. Transmission values were calibrated by the system so that a value of 100% represented a homogeneous liquid solution, with 0% representing a fully opaque solution. Data was initially analysed through the standard route (STR), where Tc was taken as the onset point where during the cooling segments the light transmittance dropped from 100% to approximately 95% and Tdiss was taken during the heating segments as the point where after a period of transmittance being at 0%, the light transmittance reached 100% and remained at this level for the remainder of the heating period. Raw transmission vs. temperature data was also reanalysed (RRT), for solutions that demonstrated two distinct regions in the profiles, one linear and one non-linear, for both the crystallisation and dissolution processes. For this analysis, crystallisation and dissolution of eicosane were represented by the linear regions, with crystallisation and dissolution of IA represented by non-linear regions. Crystallisation temperatures of eicosane were determined when laser transmission values began to decrease rapidly in a linear manner and dissolution temperatures were taken when laser transmission changed from a rapidly increasing linear region to a non-linear increasing region. RRT generated minor errors in the dissolution temperature values of eicosane, given that it was not possible to reach a transmission value of 100% prior to the change from a linear to non-linear increasing transmission region. Nevertheless, this had negligible impact on the values of eicosane dissolution temperatures, given that very rapid increases in laser transmission are associated with eicosane dissolution, thereby ensuring that the determined dissolution temperatures were very similar/the same as would have been determined with a transmission value of 100%.

The 5 crystallisation and dissolution temperature values determined for each heating/cooling rate at each solution concentration, for each IA treat rate was used to generate average values that could be used for subsequent analysis of the cooling and heating rate dependence of the crystallisation and dissolution points, respectively. This was determined by plotting Tc and Tdiss vs. heating/cooling rate (q), setting linear regressions through the data and extrapolating these regressions back to a cooling rate of 0 °C min^-1^. This enabled that the rate independent crystallisation temperature at the kinetic limit (Tc,l) and equilibrium saturation temperature (Te) to be determined, respectively. The difference between Tc,l and Te was taken as the steady-state MSZW.

A plot of Tc,l and Te vs. solution concentration was generated for the solutions containing 3 wt% IA using data collected from both RRT and STR analysis routes, in order to visually observe the impact of the different analysis routes on the collected data. Furthermore, a plot of the average steady-state MSZWs over the concentration ranges studied for a given IA treat rate vs. IA treat rate was generated for data collected through RRT and STR analysis routes to highlight this impact further over the full range of solutions studied. Turbidity vs. temperature plots were also generated for solutions with a 100 wt% IA treat rate, at a heating/cooling cycle of 0.25 °C min^-1^ to observe the non-linear dependencies of the crystallisation and dissolution processes involved for IA in toluene solution.

#### 2.3.3 List of Procedural Steps

Below is a list of steps required to reproduce the data presented within this article:

• For a given IA treat rate, prepare solutions at 4 different concentrations (including the sum of both eicosane and IA) with toluene as the solvent.
• Homogenize the solutions by heating them under agitation, until the solid is fully dissolved.
• Transfer 1 ml of the solutions to 1.5 ml vials with small magnetic stirrer bars placed in them and place them in the Technobis Crystal 16 system, with a single block containing 4 different solution concentrations for a given IA treat rate.
• Set each block to a different heating/cooling rate and set up a temperature profile to heat and cool the solutions, in order to generate crystallisation and dissolution data. Perform this profile 5 times to generate repeat data.
• Determine crystallisation (Tc) and dissolution (Tdiss) temperatures by changes in the transmission vs. temperature data generated by the Technobis Crystal 16 system and average the values, using both RRT and STR analysis routes.
• Plot average Tc and Tdiss data as a function of heating/cooling rate, fitting linear regressions through the data and extrapolate the values to a 0 rate, producing the rate independent crystallisation temperature at the kinetic limit (Tc,l) and equilibrium saturation temperature (Te), respectively.
• Plot Tc,l and Te as a function of solution concentration for both RRT and STR analysis routes to observe the impact of these routes on that data.
• Determine the steady-state MSZWs by taking the difference between Tc,l and Te.
• Average the MSZWs over the concentrations studied for a given IA treat rate and plot these values as a function of the IA treat rates, for both RRT and STR analysis routes, to observe the impact of the analysis routes on this key crystallisation metric parameter.
• Plot transmission vs. temperature data for solutions containing IA in toluene, in the absence of any eicosane, to see the non-linear dependency of the crystallisation and dissolution processes of IA, providing insight into the requirement for the RRT analysis route when studying multi-phase crystallisation systems.

## CRediT authorship contribution statement

**Peter L. Kaskiewicz:** Conceptualization, Methodology, Formal analysis, Investigation, Writing – original draft, Writing – review & editing, Visualization. **Ruth Downie:** Supervision. **Peter J. Dowding:** Supervision. **Neil George:** Supervision. **Kevin J. Roberts:** Supervision, Conceptualization, Validation, Writing – review & editing.

## Declaration of Competing Interest

The authors declare that they have no known competing financial interests or personal relationships that could have appeared to influence the work reported in this paper.

## Data Availability

Crystallisation and Dissolution Temperatures of a Model Fuel Comprising Eicosane Crystallising from Supersaturated Toluene Solutions in the Presence of a Cold-Flow Improver Additi (Original data) (White Rose Repository).
